# Setting positive end-expiratory pressure: the use of esophageal pressure measurements

**DOI:** 10.1097/MCC.0000000000001120

**Published:** 2023-12-01

**Authors:** Peter Somhorst, Amne Mousa, Annemijn H. Jonkman

**Affiliations:** a Department of Intensive Care Medicine, Erasmus Medical Center, Rotterdam, The Netherlands; b Department of Intensive Care Medicine, Amsterdam UMC, location VUmc, Amsterdam, The Netherlands

**Keywords:** esophageal manometry, mechanical ventilation, positive end-expiratory pressure, transpulmonary pressure

## Abstract

**Purpose of review:**

To summarize the key concepts, physiological rationale and clinical evidence for titrating positive end-expiratory pressure (PEEP) using transpulmonary pressure (*P*_L_) derived from esophageal manometry, and describe considerations to facilitate bedside implementation.

**Recent findings:**

The goal of an esophageal pressure-based PEEP setting is to have sufficient *P*_L_ at end-expiration to keep (part of) the lung open at the end of expiration. Although randomized studies (EPVent-1 and EPVent-2) have not yet proven a clinical benefit of this approach, a recent posthoc analysis of EPVent-2 revealed a potential benefit in patients with lower APACHE II score and when PEEP setting resulted in end-expiratory *P*_L_ values close to 0 ± 2 cmH_2_O instead of higher or more negative values. Technological advances have made esophageal pressure monitoring easier to implement at the bedside, but challenges regarding obtaining reliable measurements should be acknowledged.

**Summary:**

Esophageal pressure monitoring has the potential to individualize the PEEP settings. Future studies are needed to evaluate the clinical benefit of such approach.

## INTRODUCTION

The importance of titrating positive end-expiratory pressure (PEEP) to the individual patient's respiratory mechanics has been well recognized [1], considering the heterogeneity of acute respiratory distress syndrome (ARDS) and the large between-patient variability in response to higher pressures [2,3]. Incorporating simple bedside measurements such as plateau pressure and driving pressure provide information of global respiratory system mechanics; however, they do not inform about the distending pressures of the lungs and chest wall and the effects of PEEP on these compartments separately. Assessment of partitioned mechanics requires esophageal manometry for the measurement of esophageal pressure (*P*_es_) as surrogate for pleural pressure (*P*_pl_). For a detailed practical step-by-step approach for bedside measurement of *P*_es_ and its use in the full context of a lung-protective ventilation strategy, we refer to a recent publication [4^▪▪^]. Theoretically, a *P*_es_-guided PEEP setting could prevent atelectasis formation and enhance lung recruitment, which is of particular interest in ARDS and in patients with high *P*_Pl_ due to other causes, as for example patients with obesity [5]. In this review, we present key concepts and the physiological rationale for titrating PEEP using *P*_es_, we discuss the current clinical evidence for this approach, and provide considerations to facilitate bedside implementation. 

**Box 1:**
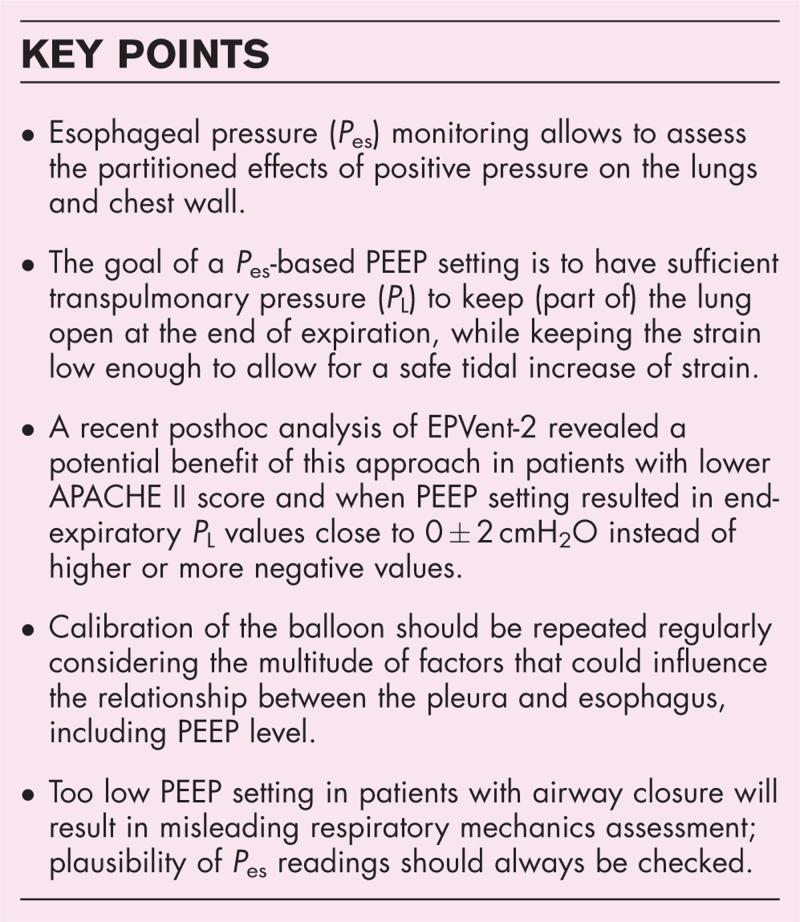
no caption available

## FROM PHYSICAL CONCEPTS TO PHYSIOLOGICAL RATIONALE

In any hollow organ, the steady state volume is dictated by its elastance and the transmural pressure. A constant positive pressure applied to the airways – the transrespiratory pressure or airway pressure, *P*_aw_ – is the transmural pressure of the respiratory system, i.e., the pressure difference between the inside of the respiratory system and surface of the body (0 cmH_2_O). *P*_aw_ can be divided into two parts that act in series. The pressure required to inflate the lung is the transpulmonary pressure, *P*_L_, which is the difference in pressure between the inside and outside of the lung tissue. The pressure required to stretch the chest wall is the transthoracic or pleural pressure, *P*_pl_, that is, the difference in pressure between the pleural space and the surface of the body (0 cmH_2_O). This can be summarized as $P_{\text{aw}}=P_{\text{L}}+P_{\text{pl}}$.

Two methods have been described to calculate *P*_L_. It can be derived from the directly measured *P*_aw_ and *P*_pl_ (*P*_L_ = *P*_aw_ – *P*_pl_, direct method) [6], or can be calculated utilizing the elastances of the lung and respiratory system (elastance-derived method) [7,8]. The elastance ratio (${\text{E}}_{\text{ratio}}={\text{E}}_{\text{lung}}/{\text{E}}_{\text{RS}}$) is defined as the elastance of the lung (*E*_lung_) divided by the elastance of the entire respiratory system (*E*_RS_) and reflects how much of the *P*_aw_ is used to inflate the lung: $P_{\text{L}}=P_{\text{aw}}\times E_{\text{ratio}}$. In healthy adults approximately 60% of the *P*_aw_ is required to inflate the lung, resulting in an *E*_ratio_ of 0.6 [9], but this ratio can be higher or lower with ARDS [10].

### Stress and strain

The elastance of the lung describes the elastic properties of both lungs, including airways. These elastic properties are influenced by, among others, the size of the lung that is actually ventilated. A smaller supple lung can have the same elastance as a larger stiff lung. Since the exact volume of the lungs is most often unknown, the lung elastance does not reflect local mechanics: a singular lung (e.g., after a pneumectomy) or ARDS ‘baby lung’ [11] have different elastic properties compared to the healthy situation. Lung tissue has a specific lung elastance that is unrelated to lung volume, but reflects the intrinsic tissue mechanical properties. The specific elastance is the transpulmonary pressure at which the lung volume at the end of a normal expiration (i.e., functional residual capacity (FRC)) doubles. For healthy lung tissue, this is around 12 cmH_2_O [10]. The associated lung deformation – i.e., the increase in volume relative to the FRC divided by the FRC – is called strain^1^. Animal studies revealed that risk of ventilator-induced lung injury (VILI) is high when the lungs are stretched beyond a strain of 2–2.5 [12,13]. Although a large part of this risk is due to dynamic strain, i.e., tidal ventilation, static strain (i.e., volume increase due to PEEP) can also contribute to lung overstretching [12]. VILI mitigation should therefore involve limiting the strain and thereby the stress (i.e., *P*_L_) during the full respiratory cycle.

### Gravity effect

Even in steady state, *P*_L_ is not constant throughout the lung but varies as a result of gravitational forces that exist due to the weight of lung tissue, and is aggravated by increased lung weight in ARDS patients [14]. In the supine position, the weight of the lung pushes on the dorsal pleurae, increasing *P*_pl_, and pulls on the ventral pleurae, decreasing *P*_pl_. Hence, *P*_L_ is less positive (or more negative) in the direction of gravity (towards the ground), and more positive (or less negative) away from the direction of gravity. Note that *P*_L_ as estimated via esophageal manometry does not include this gradient and provides a ‘global’ measure of *P*_L_. Yoshida *et al.*[15] showed experimentally that *P*_L_ as measured by the direct method mostly reflects the *P*_L_ of the dorsal part of the lung, while *P*_L_ deduced from the elastance-derived method mostly reflects *P*_L_ in the nondependent lung. The directly measured *P*_L_ also reflected dorsal *P*_pl_ in a model of asymmetrical lung injury, where *P*_pl_ equalizes between the injured and noninjured lung [16^▪^].

### *P*_L_ for positive end-expiratory pressure setting: direct method and target pressure

In the normal situation, *P*_L_ at end-expiration (*P*_L,ee_) is slightly positive, indicating a positive net pressure outward that keeps the lung open. When *P*_L,ee_ is low, bronchial collapse and atelectasis could occur. Therefore, the main goal of PEEP is to keep the *P*_L,ee_ high enough to prevent sizeable collapse. However, high *P*_L,ee_ also increases static strain, thereby increasing the risk of reaching harmful strains during tidal ventilation. The goal of a *P*_es_-based PEEP setting is to have sufficient *P*_L,ee_ to keep (part of) the lung open at the end of expiration, while keeping the strain low enough to allow for a safe tidal increase of strain.

Since the absolute values of *P*_es_ best reflect the dependent lung regions that are at highest risk for lung collapse, the direct method for *P*_L,ee_ calculation (*P*_aw_–*P*_es_ at the end of expiration using end-expiratory occlusions) for setting PEEP has been proposed. To note, PEEP strategy using the elastance-derived *P*_L,ee_ will yield different results and cannot be considered interchangeable [17].

Talmor *et al.*[6] hypothesized that a positive *P*_L,ee_ should be targeted, considering that *P*_L_ is slightly positive in a normal situation. Yoshida *et al.*[15] estimated that they required a *P*_L,ee_ of 4.6 ± 2.2 cmH_2_O to prevent all collapse in their pig model. Experimental data in swine suggest that the lung mechanical properties are at an optimal compromise where lung collapse and overdistention are jointly minimized when *P*_L,ee_ is low at 2 cmH_2_O [18].

**FIGURE 1:**
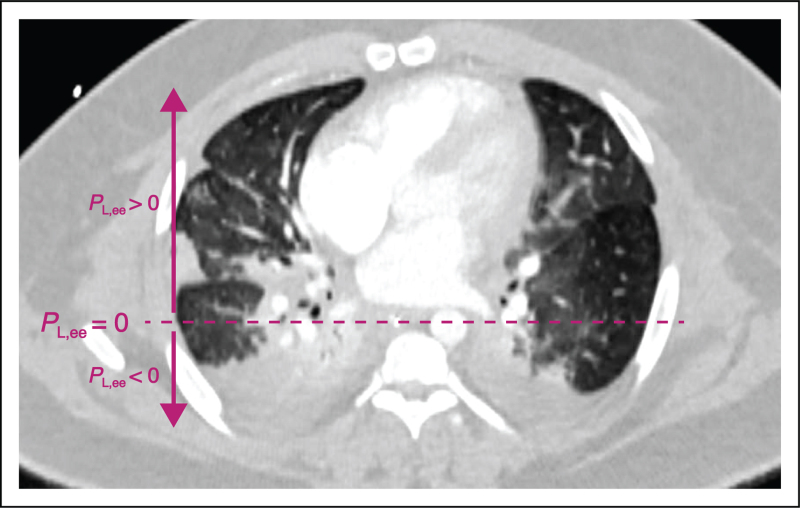
CT scan of a patient with acute respiratory distress syndrome due to COVID-19. The spatial gradient of *P*_L_ is aggravated with heterogeneous lung injury. Note that a targeted *P*_L,ee_ of 0 cmH_2_O in supine position will reflect the *P*_L_ at the level of the esophagus; the *P*_L_ will be negative in the areas dorsal to the esophagus, and positive in the ventral parts of the lungs.

Any strategy for setting PEEP results in a gradient of *P*_L_ in the thorax. Nondependent areas will always experience higher *P*_L_ and could be at risk for overdistension, while the dependent areas will always experience lower *P*_L_. In the case of a targeted *P*_L,ee_ of 0 cmH_2_O at the level of the esophagus in supine position, the *P*_L_ will be negative in the areas dorsal to the esophagus, and positive in the ventral parts of the lungs (Fig. 1).

## LATEST CLINICAL EVIDENCE FOR A *P*_es_-BASED POSITIVE END-EXPIRATORY PRESSURE SETTING

Two randomized clinical studies (EPVent-1 [6] and EPVent-2 [19]) on *P*_es_-guided PEEP in ARDS have been performed, yet clear evidence on how to best individualize PEEP using *P*_es_ is lacking. EPVent-1 was a small (*n* = 61) single center study where PEEP setting to maintain a positive *P*_L,ee_ was compared to using the low PEEP/FiO_2_ table [6]. To note, no maximum values for *P*_L,ee_ were protocolized. *P*_es_-guided PEEP resulted in higher PEEP levels (at 72 h: mean 17 vs. 10 cmH_2_O for *P*_es_-guided vs. conventional strategy). In addition, the intervention group showed better response on oxygenation (primary study endpoint; PaO_2_/FiO_2_: mean 280 vs. 191 mmHg for *P*_es_-guided vs. conventional strategy) and respiratory system compliance (mean 45 vs. 35 mL/cmH_2_O for *P*_es_-guided vs. conventional strategy) at 72 h. Because of the strong oxygenation effect, the trial was prematurely terminated. Despite not being powered on patient outcomes, a trend towards improved 28-day mortality rate was reported [6]. These results stimulated the design of the larger follow-up study EPVent-2 [19] in 200 patients with moderate-severe ARDS, which was a multicenter study powered on a composite primary endpoint including mortality and ventilator-free days at day 28; however, no benefit of a *P*_es_-guided PEEP strategy on patient outcomes was found.

Differences in outcome between both studies can be partially explained by different patient characteristics and interventions. Whereas the control group in the EPVent-1 trial received PEEP according to the lower PEEP/FiO_2_ table, resulting in much lower PEEP levels than the intervention group and also negative *P*_L,ee_ values predisposing to atelectasis [6], the comparator strategy of the EPVent-2 trial was the high PEEP/FiO_2_ table [19]. Consequently, PEEP levels (and also plateau pressures) for the control group were similar to those in the intervention group for the first week of study, on average resulting in *P*_L,ee_ ≥0 cmH_2_O values until day 3 [19], and were also higher compared to other ARDS trials [20]. High values of *P*_L,ee_ up to 6 cmH_2_O were allowed [19], putting the nondependent lung at risk of overdistension. Furthermore, the EPVent-2 trial included only moderate and severe ARDS with primarily pulmonary risk factors for ARDS [19], whereas the EPVent-1 trial also included mild ARDS and reported a large contribution of intra-abdominal risk factors for ARDS (in 40% of patients) [6].

New insights by Sarge *et al.*[21^▪▪^] after a posthoc analysis of EPVent-2 revealed that a *P*_es_-guided PEEP strategy was associated with improved survival in two conditions; this should be confirmed prospectively:

First, in patients with lower disease severity, categorized by an Acute Physiology and Chronic Health Evaluation (APACHE) II score < 27.5 (being the median value) [21^▪▪^]. They hypothesized that this could be due to the likelihood that mortality in patients with greater disease severity was less likely to be caused by pulmonary status and mechanical ventilation strategy alone [21^▪▪^].

Second, when PEEP setting resulted in *P*_L,ee_ close to 0 ± 2 cmH_2_O instead of higher or more negative values. This association was found independent of treatment group and multiorgan dysfunction severity. It is in line with the hypothesis that maintaining *P*_L,ee_ around 0 cmH_2_O most likely provides a good balance between minimizing atelectrauma and lowering the risk of hemodynamic compromise and overdistension [18].

### Importance of lung recruitability testing

Another important consideration is that both EPVent studies lacked proper assessment of PEEP responsiveness, that is, lung recruitability [22], prior to setting PEEP. In fact, setting high PEEP in patients with low recruitability has detrimental effects and should be avoided [23]. PEEP can offset high *P*_pl_ and *P*_es_ could be used to estimate this effect. The EPVent-2 trial suggested that patients had minimal lung recruitment, since airway driving pressures and transpulmonary driving pressures were not different between groups, nor between baseline and first values on protocol [19]. In contrast, the EPVent-1 intervention group demonstrated better respiratory system compliance, which was suggested to reflect higher potential for lung recruitment [6]. However, both *P*_es_ and PEEP levels as titrated with *P*_es_ are not or minimally correlated with lung recruitability [24,25] and changes in respiratory system driving pressures and compliance may not properly inform about lung recruitment [3,22].

### Obesity

Patients with obesity sometimes show high *P*_pl_ due their higher chest wall and abdominal load. Note that chest wall compliance is often not altered [26,27], but this requires *P*_es_ to assess. Especially during passive mechanical ventilation, the excess fat load could result in decreased *P*_L_ and thus lower end-expiratory lung volume, which promotes airway closure and alveolar collapse. Setting PEEP to target a positive *P*_L,ee_ in obese patients has proven to be safe in terms of hemodynamic tolerance and limiting overdistention, and resulted in improved oxygenation and decreased driving pressure [28^▪^,^29^]. Furthermore, it was associated with lower mortality in patients with BMI >40 kg/m^2^[30]. Chen *et al.*[31^▪^] recently reported a significant interaction between a positive *P*_L,ee_ (direct method) and patient outcomes (lowered 60-day mortality) in obese patients. This strengthens the hypothesis that a *P*_es_-guided PEEP strategy could be especially beneficial in the obese, which requires further study.

## HOW DO WE DO IT? *P*_es_-GUIDED POSITIVE END-EXPIRATORY PRESSURE IN PRACTICE

**FIGURE 2:**
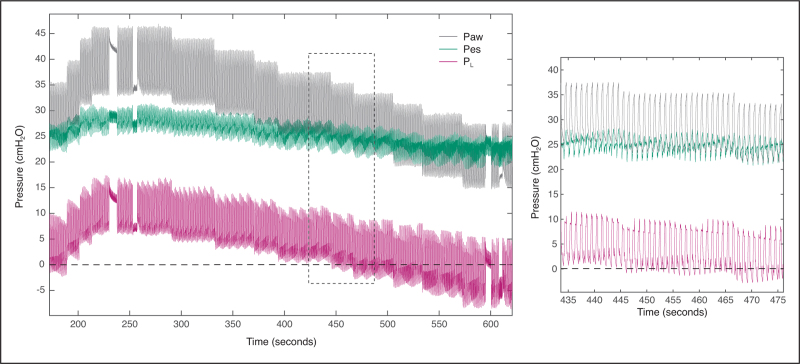
Decremental PEEP trial with synchronized recordings of airway pressure (*P*_aw_), esophageal pressure (*P*_es_) and transpulmonary pressure (*P*_L_ = *P*_aw_ – *P*_es_) during pressure-control ventilation in a critically ill patient with ARDS that was admitted to our ICU during the COVID-19 pandemic. Data are from the same patient as presented in Fig. 1. Signals were acquired at 50 Hz using dedicated equipment. PEEP was gradually increased from 23 to 32 cmH_2_O to test the patient's tolerance, before reducing PEEP in small steps. Once PEEP level was decreased below 25 cmH_2_O, *P*_L,ee_ became negative (see dotted line). ARDS, acute respiratory distress syndrome; PEEP, positive end-expiratory pressure.

**FIGURE 3:**
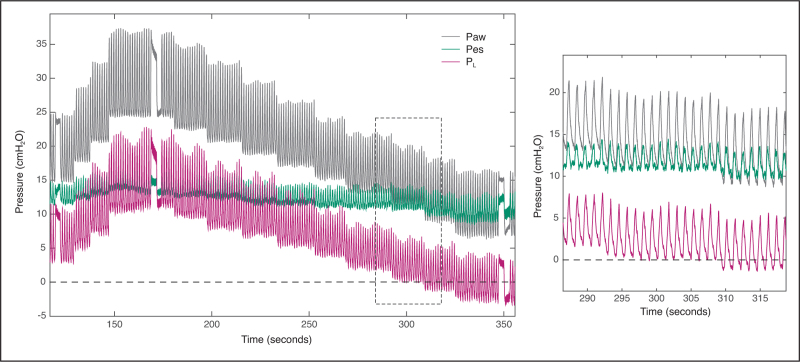
Decremental PEEP trial with synchronized recordings of airway pressure (*P*_aw_), esophageal pressure (*P*_es_) and transpulmonary pressure (*P*_L_ = *P*_aw_ – *P*_es_) during pressure-control ventilation in a critically ill patient with ARDS that was admitted to our ICU during the COVID-19 pandemic. Signals were acquired at 50 Hz using dedicated equipment. PEEP was gradually increased from 15 to 24 cmH_2_O to test the patient's tolerance, before reducing PEEP in small steps. Once PEEP level was decreased below 12 cmH_2_O, *P*_L,ee_ became negative (see dotted line). ARDS, acute respiratory distress syndrome; PEEP, positive end-expiratory pressure.

Here, we present two cases of *P*_es_ measurements during a PEEP titration (Figs. 2 and 3). *P*_aw_ was measured using a pressure sensor connected to the patient's endotracheal tube, thereby minimizing the time delay between *P*_aw_ and *P*_es_; having *P*_aw_ measurement as close as possible to the tube is important as it enhances reliability, especially during dynamic measurements. Real-time computation of *P*_L_ (using the direct method) was available at the bedside. After gradually increasing the PEEP to test the patient's tolerance to higher pressures, a decremental PEEP trial was performed. Lung hysteresis is visible, that is, note the slight increase in *P*_L,ee_ at both 15 cmH_2_O PEEP levels in Fig. 3. A *P*_es_-guided PEEP setting to reach a *P*_L,ee_ of 0 cmH_2_O would recommend a very high PEEP of 25 cmH_2_O in the patient of Fig. 2, and a moderate PEEP level of 12 cmH_2_O in Fig. 3.

## CONSIDERATIONS TO FACILITATE BEDSIDE IMPLEMENTATION

Technological advances have made Pes monitoring easier to apply at the bedside, using tools integrated within ventilator monitors or stand-alone equipment. Nevertheless, the direct method of measuring *P*_L_ for a PEEP setting strategy is limited by a number of factors influencing the relationship between the pleura and esophagus. For details and a practical approach, see [4^▪▪^]. Correct filling of the esophageal balloon is crucial, and generally it is advised to choose the filling volume at which the *P*_es_ swing during tidal ventilation is largest (*V*_best_). An underinflated balloon cannot transfer all changes in *P*_es_, while an overinflated balloon leads to stretching of the balloon itself, resulting in dampened pressure transmission [32]. Inflation of the balloon pushes the esophageal wall aside, leading to an increase in pressure inside the balloon due to the esophageal wall pressure (*P*_ew_) [33]. Several studies with different types of balloons [34,35,36^▪^] showed that careful calibration can improve reliability compared to standardized filling volumes. Importantly, these studies suggest that the filling volume based on *V*_best_ results in a overestimation of the esophageal pressure since *P*_ew_ could range from 0–8 cmH_2_O at this *V*_best_[36^▪^]. Jiang *et al.* also showed that *V*_best_ was lower in patients with a higher BMI [36^▪^]. Since the weight of the heart and mediastinum, but also body position and PEEP level can influence the measured pressure [34,37–39], calibration should be repeated regularly. The role of automated or alternative calibration methods to optimize reliability of measurements should be studied.

The plausibility of Pes readings should also be carefully checked. Peristaltic esophageal spasms or cardiac contractions can distort the Pes signal. Furthermore, *P*_L,ee_ calculation (i.e., PEEP_tot_ – end-expiratory *P*_es_) is only valid when the airways are fully open during the end-expiratory occlusion. Airway closure can be common in ARDS patients and obesity [40–43], which increases alveolar pressure and could result in misleading respiratory mechanics assessment when PEEP is set below airway opening pressure.

## CONCLUSION

The goal of an *P*_es_-based PEEP setting is to have sufficient *P*_L,ee_ to keep (part of) the lung open. Although randomized studies have not yet proven clinical benefit of this approach, a recent posthoc analysis of EPVent-2 revealed a potential benefit in patients with lower APACHE II score and when PEEP setting resulted in *P*_L,ee_ values close to 0 ± 2 cmH_2_O. Prospective studies are needed to evaluate the benefit of this approach, and should also consider recruitability assessment.

## Acknowledgements

*None.*

### Financial support and sponsorship

*None.*

### Conflicts of interest

*There are no conflicts of interest.*

## Footnotes

1 It is important to note here that strain is defined as stretch relative to an unstretched lung at FRC. One should not use the end-expiratory lung volume (EELV) when PEEP is applied to calculate the strain.

## REFERENCES AND RECOMMENDED READING

Papers of particular interest, published within the annual period of review, have been highlighted as:

▪ of special interest
▪▪ of outstanding interest
